# ODD-Net: a hybrid deep learning architecture for image dehazing

**DOI:** 10.1038/s41598-024-82558-6

**Published:** 2024-12-23

**Authors:** C. S. Asha, Abu Bakr Siddiq, Razeem Akthar, M. Ragesh Rajan, Shilpa Suresh

**Affiliations:** 1 Department of Mechatronics, Manipal Institute of Technology, Manipal Academy of Higher Education, Manipal, Karnataka 576104 India; 2 Department of Electronics and Communication Engineering, Amrita Vishwa Vidyapeetham, Amritapuri, Kerala 690525 India

**Keywords:** Image dehazing, Deep retinex, LCA, Dense depth, A Net, T Net, Engineering, Mathematics and computing

## Abstract

Haze can significantly reduce visibility and contrast of images captured outdoors, necessitating the enhancement of images. This degradation in image quality can adversely affect various applications, including autonomous driving, object detection, and surveillance, where poor visibility may lead to navigation errors and obscure crucial details. Existing dehazing techniques face several challenges: spatial methods tend to be computationally heavy, transform methods often fall short in quality, hybrid methods can be intricate and demanding, and deep learning methods require extensive datasets and computational power. To overcome these challenges, we present ODD-Net, a hybrid deep learning architecture. Our research introduces a comprehensive data set and an innovative architecture called Atmospheric Light Net (A-Net) to estimate atmospheric light, using dilated convolution, batch normalisation, and ReLU activation functions. Furthermore, we develop T-Net to measure information transmission from objects to the camera, using multiscale convolutions and nonlinear regression to create a transmission map. The integrated architecture combines pre-trained A-Net and T-Net models within the atmospheric scattering model. Comparative analysis shows that ODD-Net provides superior dehazing quality, especially in transmission map estimation and depth measurement, surpassing state-of-the-art methods such as DCP, GMAN, DehazeNet, and LCA. Our quantitative analysis reveals that ODD-Net achieves the highest performance in terms the quality metrics compared. The proposed method demonstrates notable quantitative and qualitative improvements over existing techniques, setting a new standard in image dehazing.

## Introduction

Image dehazing is crucial for enhancing the quality of images captured in outdoor settings, where haze can significantly degrade visibility and contrast^1–4^. This reduction in image quality can negatively impact various image processing applications, such as autonomous driving, object detection, and surveillance. For example, in autonomous systems, haze-induced poor visibility can result in navigation and obstacle detection errors, potentially leading to accidents. Similarly, in surveillance, haze can obscure vital details, making identification of objects or individuals difficult. Consequently, the development of effective dehazing techniques is critical to ensure the reliability and accuracy of these applications^5–8^.

This study is motivated by the need to overcome the limitations of current dehazing techniques, which include spatial methods, transform domain methods, and machine learning (ML)/deep learning (DL) approaches. Spatial methods, such as the Dark Channel Prior (DCP) and Atmospheric Scattering Model (ASM), work by estimating the transmission map to clear up images but often struggle with computational inefficiency and reduced effectiveness under complex haze conditions^1,9,10^. Transform-domain methods, such as wavelet transforms, offer computational efficiency but may not consistently deliver high-quality dehazing. Hybrid approaches seek to merge the benefits of both spatial and transform methods^11^. Meanwhile, ML/DL techniques utilise neural networks to learn dehazing patterns from data, showing considerable potential but also encountering challenges related to data requirements and computational intensity^12,13^.

He et al. proposed a dark channel prior, which involves calculating the minimum intensities over three channels within a neighbourhood. The assumption is that the dark channel for hazy images is brighter due to the white haze component, which adds equal intensities to all colour channels. This method assumes homogeneous hazing and uses the brightest 0.1% of pixels in the dark channel to estimate atmospheric light^1^. To refine the transmission map, He et al. suggested the use of soft matting, which, although effective, increases complexity and processing time. Subsequent work by He et al. demonstrated that using a guided filter instead of soft matting improves the performance of the DCP algorithm^1,14,15^. Zhu et al. introduced a color attention prior-based approach, observing that haze density increases with depth^16^. They assumed depth to be positively correlated with the difference between value and saturation in the HSV color space. Despite their effectiveness, these spatial methods often struggle with computational efficiency and may not perform well under varying atmospheric conditions. Li et al. proposed a dual domain method that adjusts the global contrast in the spatial domain for dehazing and then enhances the local contrast in the frequency domain^17,18^. This approach has shown promising results in terms of both efficiency and dehazing performance^19^.

Transform-based techniques such as Fourier and wavelet transforms have been employed to distinguish between hazy and clear images. Ancuti et al. introduced a wavelet-based dehazing approach that uses the multiscale nature of wavelet transforms to enhance the visibility of hazy images^10,20,21^. However, the authors noted that, while wavelet-based methods offer computational efficiency, they may not consistently deliver high-quality dehazing results, especially in complex hazy scenarios. Tarel and Hautière proposed a Fourier-based dehazing method that operates in the frequency domain^11^. By modelling the haze as a low-pass filter, the authors were able to remove the haze component and restore the clear scene. The multilevel wavelet transforms and Haar wavelet transforms, combined with regularised optimisation, enhance dehazing efficacy by applying optimisation to low-frequency subband decomposition. These techniques are known for their efficiency and durability, making them suitable for real-world applications. However, they may not always achieve the high-quality results required for complex haze conditions^22,23,24^.

Hybrid techniques blend spatial and transform-based methodologies to improve image quality. The application of guided anisotropic diffusion and iterative learning-based image filters (GADILF) enhances the transmission map, reduces color and texture distortion. Similarly, Meng et al. introduced a method that uses both dark channel prior and wavelet-based processing to achieve efficient and effective dehazing^12^. While the hybrid approach aimed to leverage the strengths of both spatial and transform-based techniques, the authors acknowledged the need for further improvements in handling complex hazy scenes^25,26^. Despite these improvements, hybrid methods can be computationally heavy and may not always balance performance and efficiency effectively^13–15,27–29^.

The development of DL techniques has significantly advanced dehazing methods^30^. Convolutional Neural Networks (CNNs) have been used to map hazy images to haze clear images. AOD-Net^31^ and its variants^32^, estimate the transmission map and atmospheric light simultaneously, providing a more sophisticated architecture for dehazing. FFA-Net^28^ uses a feature fusion architecture to enhance the dehazing performance, although it is computationally intensive^28^. Pavan et al.^33^ proposed a light convolution network focused on time and computational efficiency for real-time applications. However, it struggles with low light and thick haze conditions. Other notable methods include the Generic Model-Agnostic Convolutional Neural Network (GMAN) for single image dehazing by Liu et al.^34^, and the Gated Context Aggregation (GCA) Network by Chen et al.^32,35^, which uses smoothed dilation and a gated sub-network to remove hazy elements and artifacts effectively. Major research was initiated by Singh et al. using different neural network-based architectures for single image dehazing^29,36–39^. Despite these advancements, challenges remain in achieving accurate representation and reconstruction under diverse haze conditions.

In recent years, significant progress has been made in developing spatial, transform-based, hybrid, and ML/DL-based techniques for single image dehazing. Each method offers unique strengths and faces specific challenges. Spatial methods are often computationally intensive, transform methods may not achieve high-quality results, hybrid methods can be complex and computationally heavy, and ML/DL methods require large datasets and significant computational resources. The performance validation is more commonly based on full reference metrics as per the literature, and not practical for real-time applications. Addressing these gaps is crucial to develop more robust and efficient dehazing techniques capable of handling various types of haze and atmospheric conditions^27,30,40^. The major contributions of this study are

- A new data set is developed that addresses various challenges associated with haze, providing a comprehensive resource for dehazing research.
- An architecture termed as Atmospheric Light Net (A-net) is modelled for atmospheric light estimation inspired from LCA-net, integrating dilated convolution, batch normalization, and ReLU activation functions to expedite convergence and feature learning.
- A novel model, T Net, is developed to quantify the amount of information transmitted from objects to the camera. Leveraging early approaches like DehazeNet, T Net employs multiscale convolutions and nonlinear regression to generate a transmission map, which correlates with the image’s depth.
- An end-to-end architecture is developed by pre-trained A-Net and T-Net models using the atmospheric scattering model, resulting in the creation of ODD-Net (Outdoor Depth-based Dehazing Model).

The remainder of the paper is structured as follows. Section "Background study" provides a comprehensive background study, covering various base models and the approach taken for dataset creation. In Sect. “Proposed model (Outdoor depth-based dehazing network)”, the proposed model is detailed and discusses its architecture and methodology. Section "Results and discussion" presents the results and discussions, including discussions on assumptions, experimental setup, quantitative and qualitative comparisons. Finally, Sect. “Conclusions” concludes by providing insights into future directions of research in the field.

## Background study

### Atmospheric scattering model

The presence of haze in an image can be attributed to two primary factors, viz, direct attenuation: which occurs due to the decay of light as it passes through the medium and air light, which results from the scattering of light in the atmosphere. The atmospheric scattering model is expressed mathematically as *I(x)* = *J (x)t(x)* + *A(1 − t(x))*, where *I(x)* represents the hazy image, *J (x)* denotes the haze free image or scene radiance, A is the global atmospheric light, and *t(x)* is the transmission of the medium. It indicates the fraction of light that is not scattered and is defined as t(x) = e − β.d(x), where β is the scattering coefficient of the atmosphere, and *d(x)* is the distance from the point of the scene to the camera. This equation illustrates that scene radiance is attenuated exponentially with the increase in scene depth. The model operates under the assumption of homogeneous scattering of light and a constant scattering coefficient throughout the medium.

### Retinex model

The Retinex model is based on human visual perception theory and aims to explain how the human eye perceives colours under varying lighting conditions^41^. This model assumes that an observed image *I(x)* can be decomposed into the product of two components: the reflectance *R(x)* and the illumination *L(x).* Mathematically, it can be expressed as *I(x)* = *R(x).L(x),* where *I(x)* is the observed image, *R(x)* represents the reflectance (intrinsic colour of objects), and *L(x)* denotes the illumination (lighting conditions). The goal of the Retinex model in dehazing is to estimate the reflectance *R(x)* from the observed image *I(x)* by appropriately separating the effects of illumination.

To achieve this separation, the model often employs logarithmic transformations to convert the multiplicative relationship into an additive one, making it easier to handle. Taking the logarithm of both sides of the equation:

$$log(I(x))=log(R(x))+log(L(x))$$

Let $${\varvec{i}}({\varvec{x}})={\varvec{l}}{\varvec{o}}\boldsymbol{ }{\varvec{g}}({\varvec{I}}({\varvec{x}})),\boldsymbol{ }\boldsymbol{ }{\varvec{r}}({\varvec{x}})={\varvec{l}}{\varvec{o}}\boldsymbol{ }{\varvec{g}}({\varvec{R}}({\varvec{x}})){\varvec{a}}{\varvec{n}}{\varvec{d}}\boldsymbol{ }{\varvec{l}}({\varvec{x}})=\mathbf{l}\mathbf{o}\mathbf{g}({\varvec{L}}({\varvec{x}}))$$

The equation then becomes $\mathbf{i}(\mathbf{x})=\mathbf{r}(\mathbf{x})+\mathbf{l}(\mathbf{x})$

The challenge lies in estimating *r(x)* and I*(x)* from *i(x).* Various algorithms, such as single-scale retinex (SSR) and multi-scale retinex (MSR), have been developed to perform this decomposition^42,43^. The Deep Retinex model comprises two key components: the Residual Illumination Map Estimation Network (RIMEN) and Channel and Spatial Attention Dehazing U-Net (CASDUN). RIMEN is responsible for estimating the residual illumination map, which contains crucial local and global information for haze removal. It utilizes a multi-scale subnetwork with three branches, each processing input hazy images at different scales to capture both global context and local details effectively. Each branch consists of a down sampling layer, a residual dense block (RDB), and a transpose convolution layer for multi-scale feature extraction^44^. The RDB employs hierarchical feature fusion and residual learning to enhance network representation. After multi-scale feature learning, the feature maps are up sampled and concatenated with the hazy image for residual illumination map prediction. On the other hand, CASDUN is a four-stage U-Net designed to capture both contextual and spatial information for haze removal that incorporates RDBs for feature extraction in each stage.

**Fig. 1:**
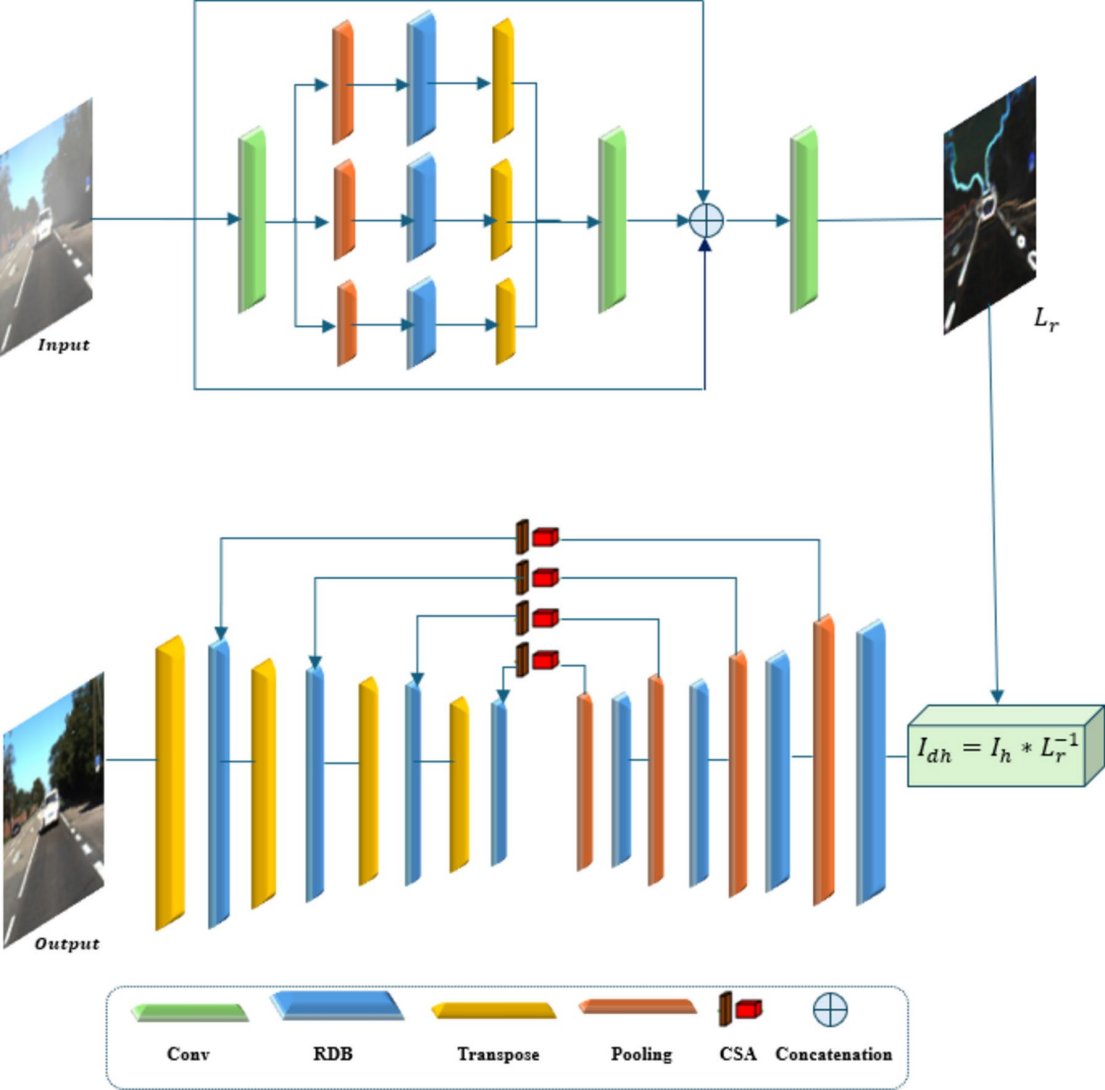
Deep retinex model.

The entire Deep Retinex model is trained end-to-end using optimization techniques, including an illumination error loss function and a dehazed image error loss function, combining pixel-wise absolute loss, SSIM loss, and smooth loss to improve performance and accuracy^44^. The block diagram is shown in Fig. 1.

### Light convolution autoencoder

Light Convolution Autoencoder (LCA) net is a light network which focuses on being faster rather than performance. The model is a sequential one, with the first part of the model acting as an encoder i.e. the image is reduced to a feature vector and then a decoder network is used to recover the haze free image from the feature vector.

**Fig. 2:**
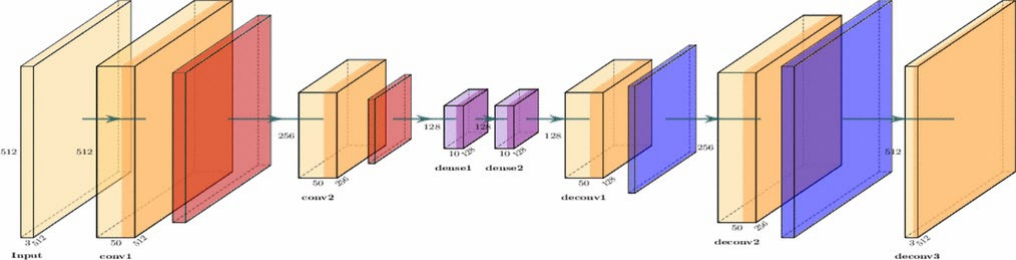
Light convolution autoencoder (LCA) Net.

The model has around 53 thousand trainable parameters. After training the model for 50 epochs taking a 30% random subset of Hazy Kitti dataset, we achieved a mean square error of 0.0056 and the model had converged giving very decent results for a simple model. The recovered image retains a small amount of haze with blurring effect. Figure 2 and Table 1 show the architecture of the LCA net.

**Table 1:** LCA Net architecture.

Layer	Output shape	Trainable parameters
Input_layer	(Batch, 352, 1216, 3)	0
Conv2d_1(encoder)	(Batch, 352, 1216, 50)	1400
Average_pool_1 (encoder)	(Batch, 176, 608, 50)	0
Conv2d_2(encoder)	(Batch, 176, 608, 50)	22,550
Average_pool_2 (encoder)	(Batch, 88, 304, 50)	0
Dense_1 (encoder)	(Batch, 88, 304, 10)	510
Dense_2 (encoder)	(Batch, 88, 304, 10)	110
Conv2d_Transpose_1 (decoder)	(Batch, 88, 304, 50)	4550
UpSample2d_1 (decoder)	(Batch, 176, 608, 50)	0
Conv2d_Transpose_2 (decoder)	(Batch, 176, 608, 50)	22,550
UpSample2d_2 (decoder)	(Batch, 352, 1216, 50)	0
Conv2d_Transpose_3 (decoder)	(Batch, 352, 1216, 3)	1353

### Dataset creation

For the creation of data set, the HazeRD dataset model was adopted, incorporating specific modifications to enhance realism and diversity in simulated haze conditions^45^. The haze simulation model in the HazeRD involves several key steps to accurately replicate hazy conditions in images. First, the haze-free images and their corresponding depth maps are considered. KITTI (https://www.cvlibs.net/datasets/kitti/raw_data.php) and RESIDE (https://sites.google.com/view/reside-dehaze-datasets/reside-standard?authuser=3D0) datasets are used for the same. The transmission map, which describes how much light can pass through the atmosphere, is calculated using the scattering parameter β, which is determined based on different weather conditions. The parameter β is related to the visible range ${R}_{m}$ in the equation β $=-\text{ln}\left(\epsilon \right) /{R}_{m}$ where ϵ represents the minimum contrast threshold visible to the human eye.

The Haze simulation considers the irradiance ${E}_{C} (x,y)$ (light received by the camera without haze) and airlight ACA_CAC (ambient light scattered into the camera). The intensity of the captured image at each channel is calculated using ${I}_{C}(x,y) ={E}_{C} (x,y)t(x,y) +{A}_{C}(x,y)(1-t(x,y))$ where $(x,y)={e}^{-\beta d(x,y)}$ . Post processing includes converting these images back to the sRGB color space after gamma correction and white balancing. This model simulates five weather conditions, from light fog to dense fog, providing varied hazy images to effectively test dehazing algorithms^45^.

**Fig. 3:**
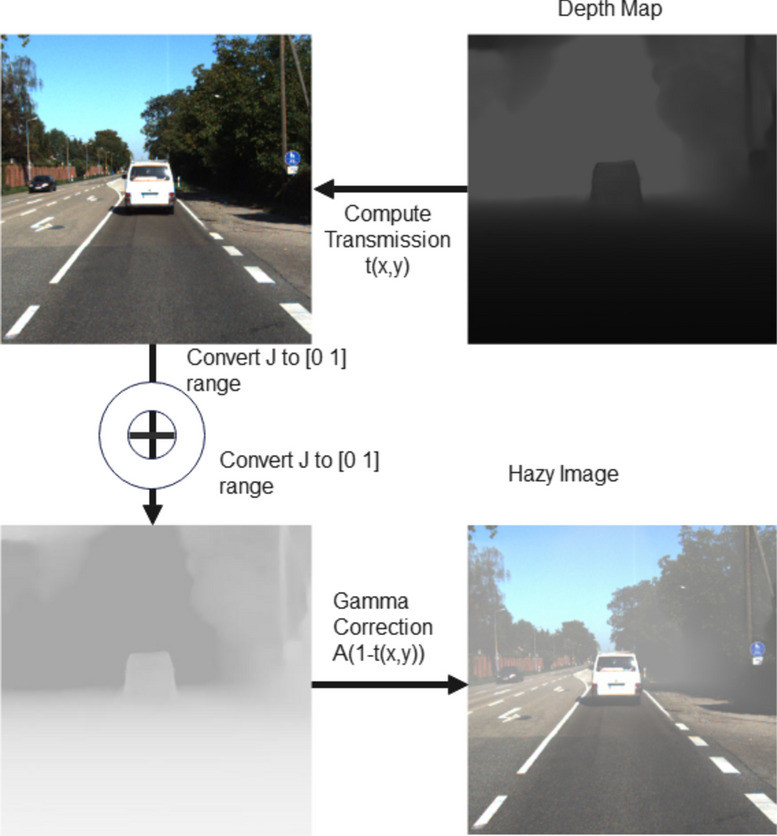
Dataset synthesis using HazeRD.

The HazeRD paper suggests setting atmospheric light at 0.76 and determining the scattering coefficient β based on the visual range, which measures the distance of clear visibility in hazy environments. For our dataset, we opted to draw atmospheric light values from a uniform distribution, while visual range values were taken from a skewed Gaussian distribution to prioritise dense-haze conditions. These distributions were optimised through visual inspections and evaluations of natural and haze-sensitive features. We used KITTI dataset for ground-truth images, captured using 1.4 megapixel Point Grey Flea 2 cameras, and LiDAR data is collected using Velodyne HDL-64E sensor, which were then converted to dense depth maps using inpainting algorithms from the NYU V2 toolbox. From the KITTI dataset, we selected 1000 images, creating five variations for each by sampling visual range values from skewed Gaussian distribution and atmospheric light values from uniform distribution. This resulted in a total of 5,000 training images. Additionally, we generated 77 test images from a different subset of the KITTI dataset^46^. Figure 3 depicts the process of dataset synthesis adopted.

## Proposed model (Outdoor depth-based dehazing network)

Our proposed model, named the outdoor depth-based dehazing network, builds upon the approach suggested in the deep retinex network. Inspired by the idea of breaking down complex problems into smaller, manageable components. We have subdivided the dehazing task into two primary challenges: atmospheric light estimation and transmission map generation. After thorough experimentation with various methodologies, we opted for this approach due to its robust theoretical foundation. A key factor in our methodology’s success was our meticulous dataset synthesis process. During this phase, we took care to include atmospheric light information to generate hazy images. Additionally, we store transmission maps corresponding to different parameters of the visual range. These measures facilitated the training of our network for transmission map generation, ensuring the effectiveness and adaptability of our proposed model.

### Atmospheric light net (A-net)

The proposed end-to-end network is inspired from LCA-net for calculating atmosphere light. We trained the network over 30 iterations, using a loss function called mean squared error (MSE). The validation results, with a loss of 0.0191, demonstrate that our model surpasses previous approaches.

**Fig. 4:**
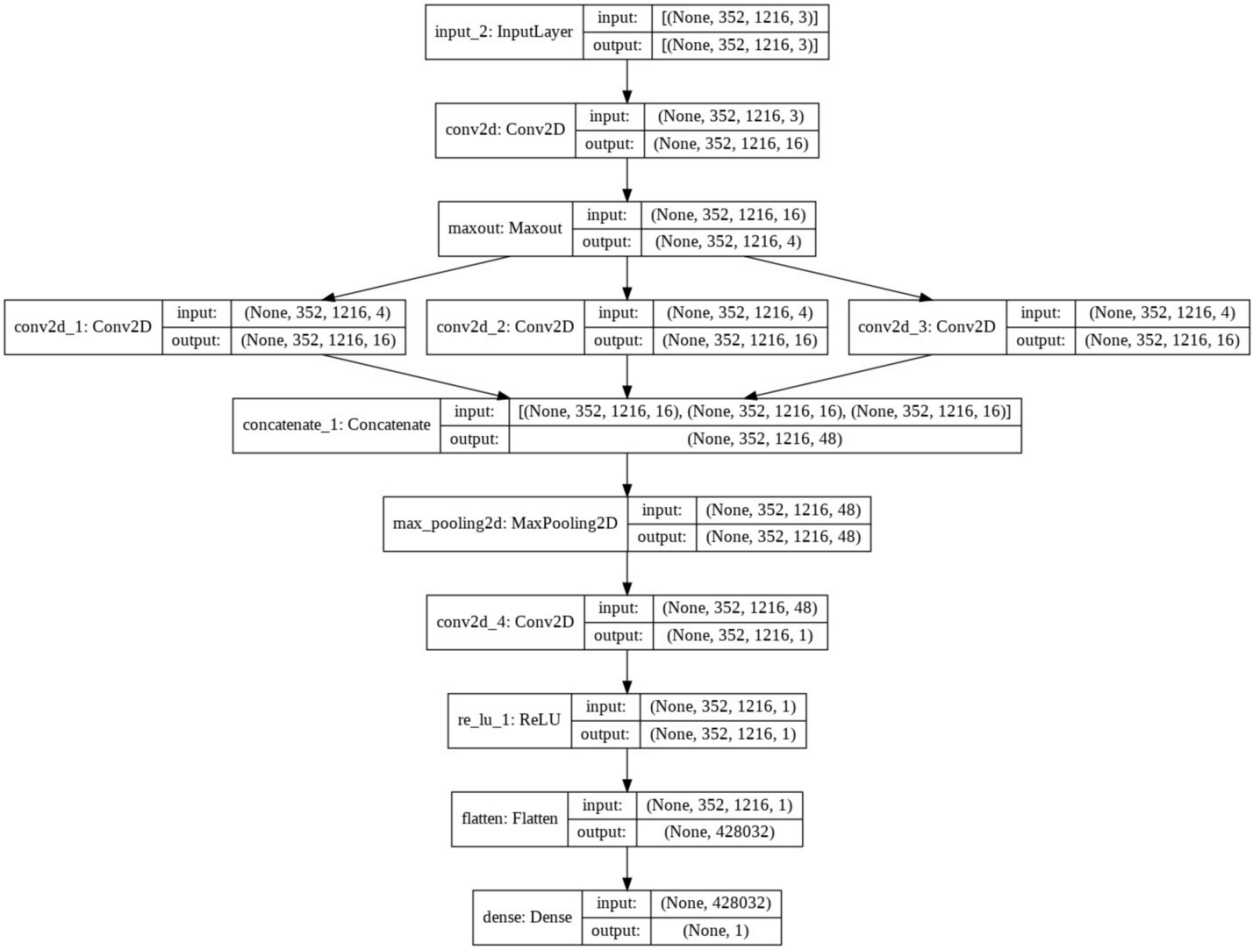
A-Net architecture.

To enhance convergence speed and effective feature learning, our network incorporates dilated convolution and batch normalization techniques. We also utilize ReLU activation functions to maintain continuous features in the output, crucial for preserving the nuances of the input image. To reduce model complexity, we employ pooling layers that compress information by selecting maximum values within sliding windows. This compression helps to manage the size of the model without sacrificing the performance. The proposed architecture is modelled with a keen focus on the atmospheric light features outlined by He et al.^1^. Specifically, we noted that atmospheric light is often the maximum intensity in both the dark channel and the input image. To capture this characteristic, we designed convolutional layers followed by max-pooling and embedded dilation layers to expand the network’s receptive field. MSE loss is utilized as objective function to effectively preserve important features of the input image, making significant strides in image dehazing research. The architectural specifications of A Net is given in Table 2 and Fig. 4.

**Table 2:** A-Net architecture.

Layer	Output shape	Parameters
Input	(Batch, 352, 1216, 3)	0 max
pooling2d	(Batch, 88, 304, 3)	0
conv2d	(Batch, 84, 300, 32)	896
max pooling2d 1	(Batch, 21, 75, 32)	0
conv2d 1	(Batch, 19, 73, 16)	4624
max pooling2d 2	(Batch, 4, 18, 16)	0
conv2d 2	(Batch, 2, 16, 8)	1160
flatten	(Batch, 256)	0
dense	(Batch, 1)	257

### Transmission map net (T-Net)

The amount of information that goes to the camera is reflected in T-Net. This value is in the range of 0 to 1, where 1 indicates a clear image and 0 implies a very dense haze. Earlier methods, such as DehazeNet, created a transmission map that correlates with the depth of image using multiscale convolutions and nonlinear regression.

We incorporated models for depth prediction in our system, specifically using monocular depth estimation models from the KITTI dataset (https://www.cvlibs.net/datasets/kitti/raw_data.php) . An appropriate model can provide enhanced depth maps and be flexible enough to accommodate modifications in the future. We examined several models, such as DenseDepth, Adabins, MonoDepth, and MonoDepth2, but finally DenseDepth is employed as our fundamental model due to its scalability and ease of use.

**Fig. 5:**
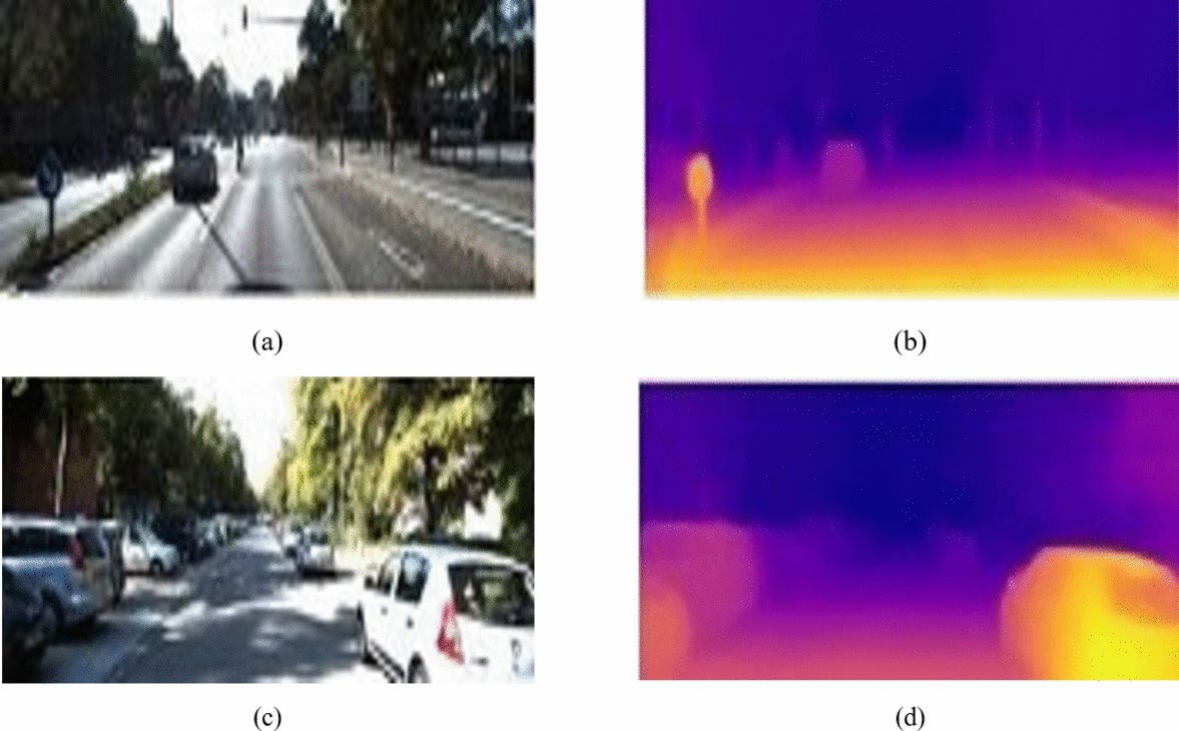
T Net output represents the transmission map from an input image (**a**) input image (**b**) transmission map.

For our scenario, DenseDepth was selected as it offered good depth map generation accuracy. Figure 5 shows the depth maps generated from DenseDepth. We adjusted DenseDepth to better suit our requirements, such as adding an upscaling block to match the output resolution with input image and including constrained ReLU activation functions to support transmission map values between 0 and 1.

**Fig. 6:**
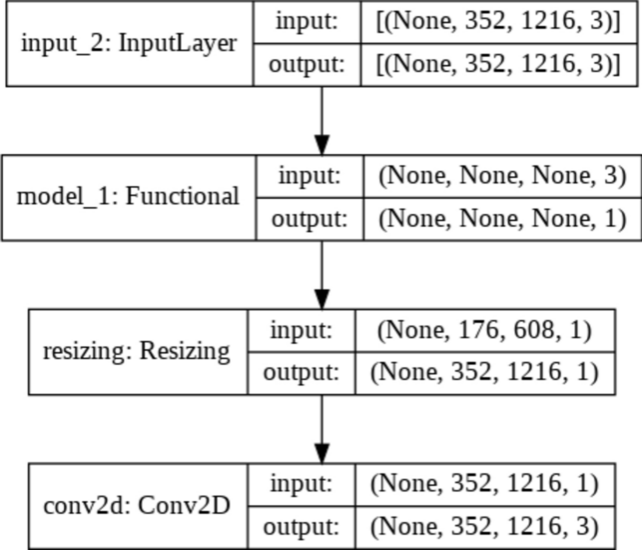
T-Net architecture.

**Fig. 7:**
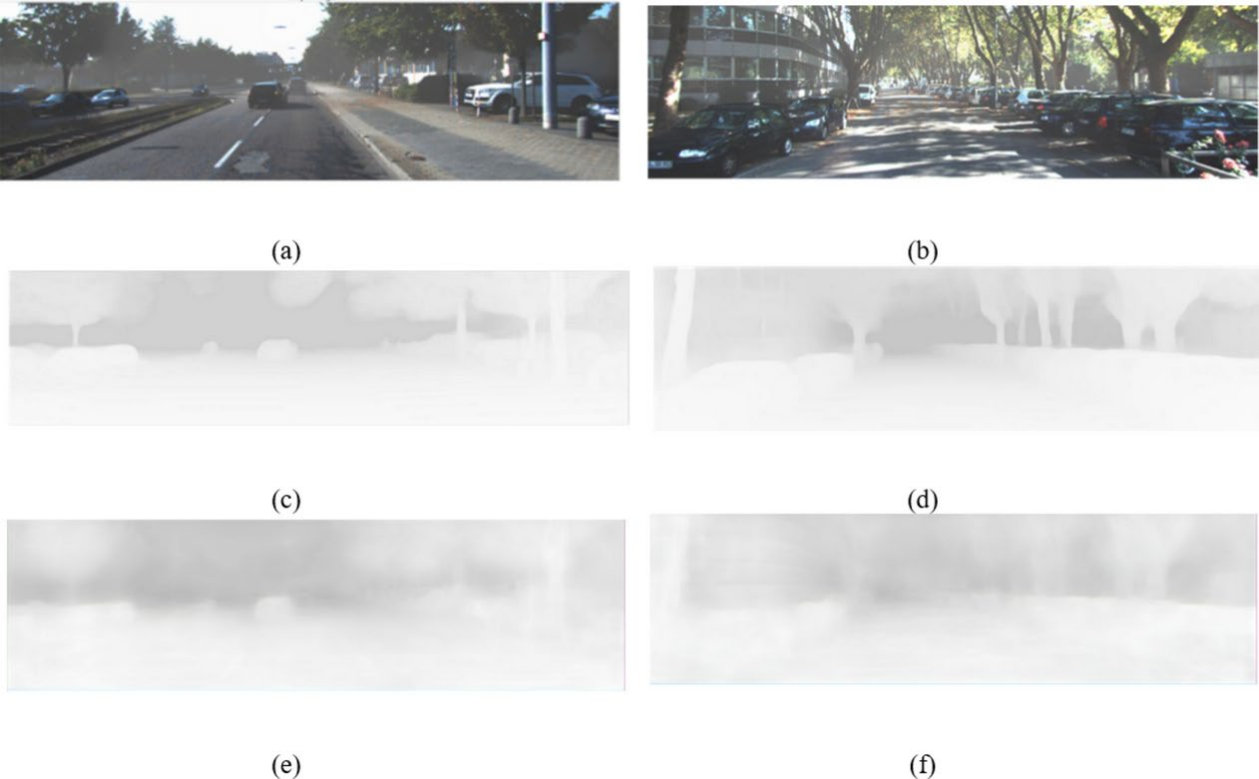
T Net Outputs: (**a**), (**b**) Hazy image (**c**), (**d**) Groundtruth T map (**e**), (**f**) Generated T map.

We further experimented with several improvements, such as pixel and channel attention blocks, and found that the EfficientNet B4 functioned satisfactorily in training, and that the Dense Net 169 encoder performed better than the Dense Net 201 encoder. These findings further supported our decision to use DenseDepth for our T-Net architecture. The T-Net architecture is presented in Figs. 6 and 7 display the responses of the T-Net architecture.

### Combination net: outdoor depth-based dehazing (ODD-Net)

**Fig. 8:**
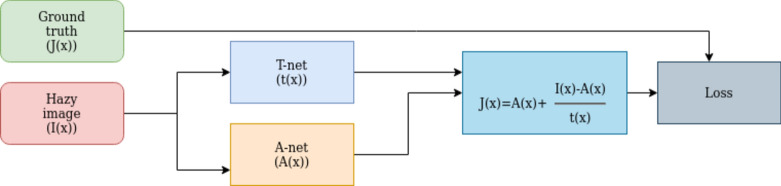
ODD net architecture.

**Fig. 9:**
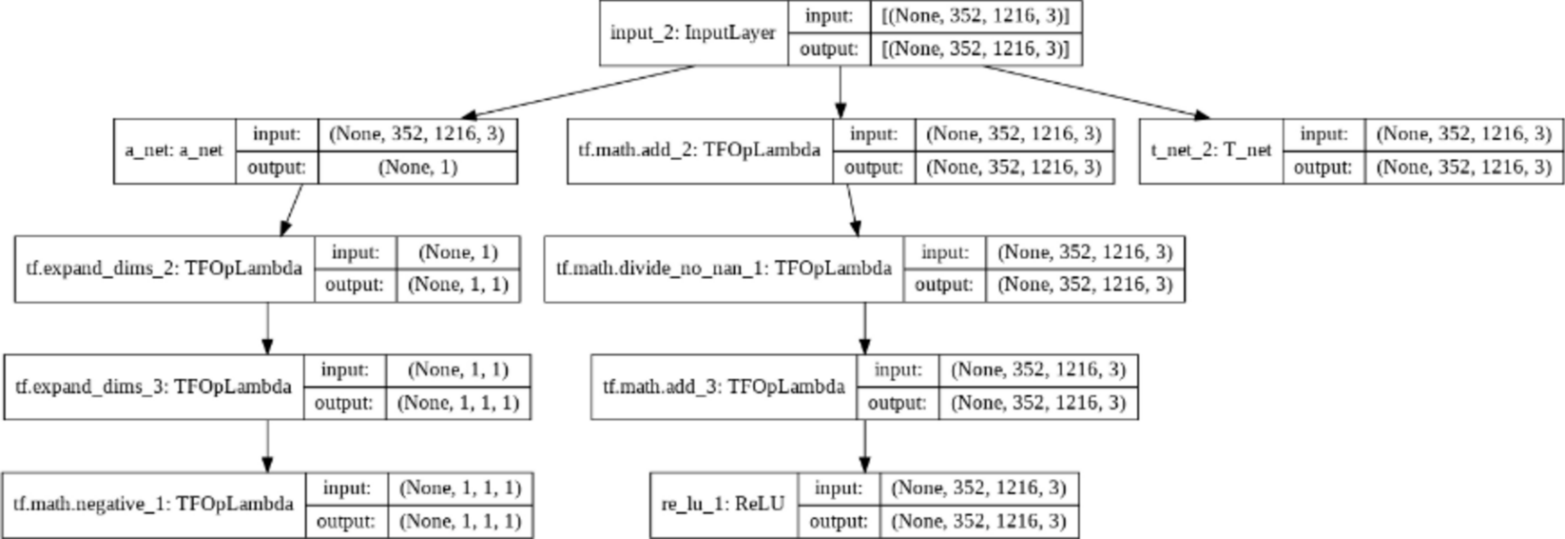
The proposed outdoor depth-based dehazing (ODD-Net) architecture.

ODD-Net is designed to integrate the outputs from both A-Net and T-Net, leveraging the strengths of each to enhance dehazing performance. The approach involved merging the intermediate outputs from A-Net and T-Net using the atmospheric scattering model. This model effectively combines the estimated atmospheric light and the transmission map to reconstruct the dehazed image. In this configuration, atmospheric light, calculated by A-Net, provides crucial information about the intensity and colour of the haze, while the transmission map from T-Net details the amount of haze that affects each pixel. By integrating these outputs, ODD-Net can accurately remove haze while preserving essential image details. To further refine the combination process, we utilized additional layers of convolution and batch normalization, ensuring smooth transitions and enhancing feature learning. This dual network synergy in ODD-Net results in superior dehazing capabilities, as demonstrated by our comparative evaluations. The architecture specifications and detailed implementation of ODD-Net are illustrated in Figs. 8 and 9.

This combined architecture, termed ODD-Net (outdoor depth-based dehazing model), was trained for 30 epochs. During training, we gradually decreased the learning rate from 1 × 10e^−4^ to 1 × 10e^−9^. Our training process used a combination of Mean Squared Error (MSE) and VGG loss functions. On completion, we achieved an MSE error of 0.0004.

## Results and discussion

The performance of the proposed method is evaluated against several recent state-of-the-art dehazing algorithms. These include LCA-Net^47^, Dehazenet^3,48^, Guided Multi-Model Adaptive Network (GMAN)^49^, Confidence Prior Model (CP)^50^, UR-Net^51^, and FFA-Net^52^. Both quantitative and qualitative results of these comparisons are detailed in Sects. "Quantitative analysis" and "Qualitative analysis".

### Experimental setup

We used NumPy, Matplotlib, and TensorFlow for our implementation. NumPy is a powerful library for numerical computation that is known for its effectiveness and speed. The visualisation tool, Matplotlib, allowed us to view input and output images. Our preferred framework was TensorFlow, an open-source toolkit used for deep learning and machine learning. Flexibility is provided by TensorFlow, especially when used with Google Colab. TensorFlow is preferred as it integrates seamlessly with Google Colab and offers access to GPU and TPU resources for faster training. We used subclassing to define our models inside the TensorFlow framework, which gave us more control over the architecture and customisation of the models. The laptop we used to run the software had an i9 processor, an RTX Nvidia 3050 graphics card and 8 GB of RAM.

### Assumptions

#### T-Net training

**Fig. 10:**
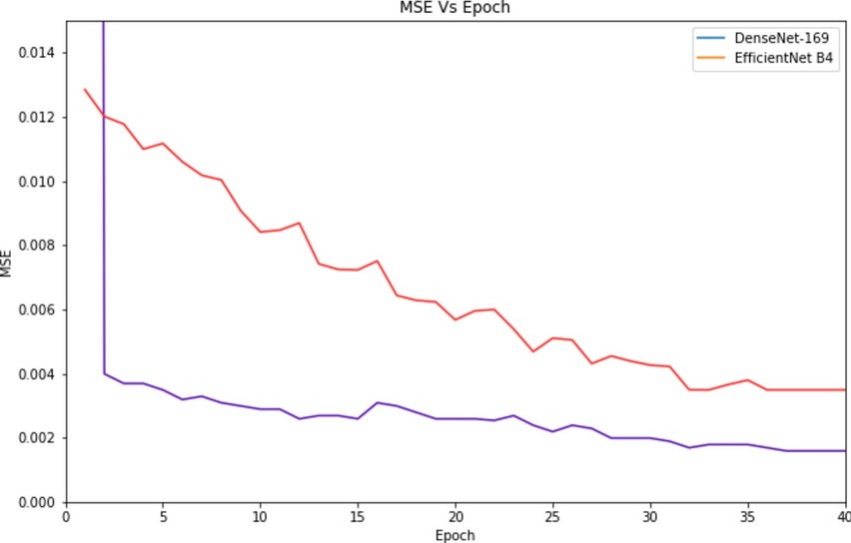
MSE vs. iterations response comparison on DenseNet and EfficientNet employed in T Net architecture.

There are 53,000 trainable parameters in T-Net. A 30% random subset of the Hazy KITTI dataset was used to train the model over 30 epochs. The convergent model yielded a mean square error of 0.0002 as validation loss and 0.0022 as training loss. The proposed end-to-end network for estimating atmospheric light is modelled using LCA-Net. MSE vs. iterations response comparison on of DenseNet and EfficientNet is shown in Fig. 10.

This study focusses on visibility outdoors, specifically how increasing air pollution reduces visibility. Our primary focus was on road conditions in order to solve this. As a result, we used a dataset that was based on the KITTI dataset, which is made up of information collected by cameras and LiDAR sensors that are installed on automobiles. To aid in visual scrutiny of the outcomes, we purposefully skewed the data set during synthesis in the direction of dense haze. Furthermore, the atmospheric scattering model, upon which our suggested model is based, is backed by strong theoretical precepts. Since outdoor images usually have a single dominant light source, this model functions under the assumptions that ambient light stays constant throughout the image and that depth is directly related to the quantity of haze present.

#### ODD net training

**Fig. 11:**
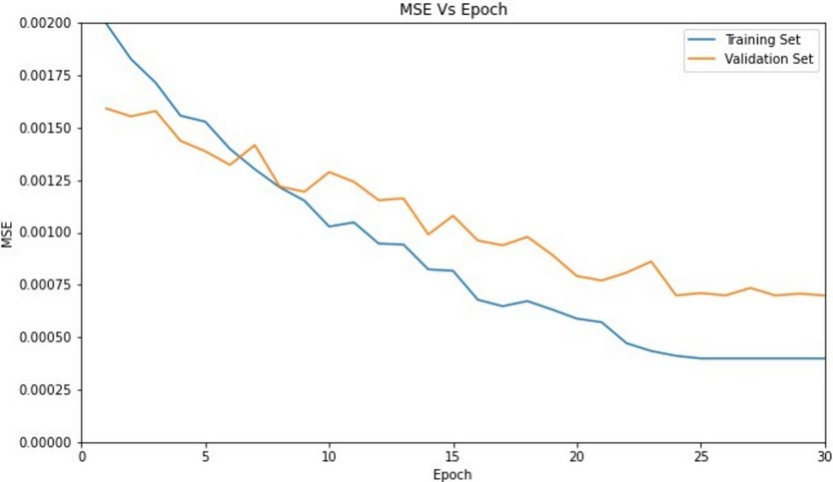
ODD net training.

This integrated architecture was trained over 30 epochs and is known as ODD-Net (outdoor depth-based dehazing model). We reduced the learning rate from 1 × 10e−4 to 1 x 10e−9 over the course of training. We used both Mean Squared Error (MSE) and VGG loss functions in our training method. had an MSE of 0.0004. The MSE loss curve for the proposed dehazing model is shown in Fig. 11. The training and validation data sets make up the data set. Variation of MSE using each component models as a part of abalation study experiment is depicted in Table 3.

**Table 3:** Ablation study results.

No.of iterations	MSE (A-Net)	MSE (T-Net)	MSE (ODD-Net)
30	0.0191	0.002	0.0004

### Quantitative analysis

KITTI, a synthetic dataset, is used to train and assess the proposed network. Since ground-truth images are available, we conduct qualitative and quantitative assessments. The peak signal-to-noise ratio, or PSNR, examines the relationship between a signal’s maximum potential power and the amount of corrupting noise that can damage its quality (Table 4). It is frequently reported in dB and used to evaluate the quality of reconstruction in movies and images following lossy compression. PSNR can be computed using logarithmic formulas and is quantified by mean squared error (MSE). The quality of the compressed or reconstructed image is better and lower error levels are indicated by a higher PSNR value. The mathematical formulas for computing mean squared error is ${\varvec{M}}{\varvec{S}}{\varvec{E}}=\frac{1}{{\varvec{m}}{\varvec{n}}}\sum_{{\varvec{i}}=1}^{{\varvec{m}}}\sum_{{\varvec{j}}=1}^{{\varvec{n}}}{({\varvec{I}}({\varvec{i}},{\varvec{j}})-{\varvec{K}}({\varvec{i}},{\varvec{j}}))}^{2}$ where ${\varvec{I}}({\varvec{i}},{\varvec{j}})$ is the pixel value of the groundtruth image, and ${\varvec{K}}({\varvec{i}},{\varvec{j}})$ is the pixel value of the original image. ${\varvec{P}}{\varvec{S}}{\varvec{N}}{\varvec{R}}=10{\mathbf{log}}_{10}\frac{{{\varvec{R}}}^{2}}{{\varvec{M}}{\varvec{S}}{\varvec{E}}}\boldsymbol{}$ where *R* is the maximum pixel value.

**Table 4:** Comparison of dehazing models on RESIDE Dataset^54^.

Metrics	LCA-Net^47^	DehazeNet^3,48^	GMAN^49^	CP^50^	U-Net^51^	FFA-Net^52^	Proposed
PSNR	12.21	17.07	17.23	14.80	19.28	20.13	20.67
SSIM	0.61	0.65	0.66	0.64	0.73	0.77	0.79
FADE	0.38	0.95	0.50	0.65	0.68	0.91	1.24
NQIE	2.85	4.42	2.93	2.70	3.71	2.7	2.67
CEIQ	3.19	3.27	3.33	3.22	3.4	3.22	3.42

A metric called the Structural Similarity Index (SSIM) is used to compare two photographs by considering aspects including brightness, contrast, and structural differences. A perceptual metric called SSIM measures how much processing, like data compression, degrades the quality of images. Since SSIM focusses on the image’s evident structures rather than noise levels like PSNR does, it is a more accurate measure of image quality degradation. The SSIM index is computed using ${\varvec{S}}{\varvec{S}}{\varvec{I}}{\varvec{M}}({\varvec{x}},{\varvec{y}})=\frac{(2\boldsymbol{ }{{\varvec{\mu}}}_{{\varvec{x}}}\boldsymbol{ }{{\varvec{\mu}}}_{{\varvec{y}}}+{{\varvec{C}}}_{1})(2\boldsymbol{ }{{\varvec{\sigma}}}_{{\varvec{x}}{\varvec{y}}}+{{\varvec{C}}}_{2})}{({{\varvec{\mu}}}_{{\varvec{x}}}^{2}+{{\varvec{\mu}}}_{{\varvec{y}}}^{2}+{{\varvec{C}}}_{1})({{\varvec{\sigma}}}_{{\varvec{x}}}^{2}+{{\varvec{\sigma}}}_{{\varvec{y}}}^{2}+{{\varvec{C}}}_{2})}$ where *x* and *y* are the image patches being compared, ${{\varvec{\mu}}}_{{\varvec{x}}}$ and ${{\varvec{\mu}}}_{{\varvec{y}}}$ are the mean values of *x* and *y* respectively, ${{\varvec{\sigma}}}_{{\varvec{x}}}^{2}$ and ${\sigma }_{y}^{2}$ are the variances of *x* and *y* respectively, ${\sigma }_{xy}$ is the covariance between *x* and *y*, ${C}_{1}$ and ${C}_{2}$ are small constants used to avoid division by zero and stabilize the calculation.

SSIM requires two images for comparison: a reference image and a processed image, typically obtained by compression or other processing methods. It is widely used in the video industry and photography to assess image quality. The performance of the image dehazing algorithm is evaluated using the FADE metric. The residual haze level in the dehazed image is measured, and a lower FADE value corresponds to improved visibility enhancement. Twelve features associated with haze are extracted from the test image and fitted to a haze-line model to compute the FADE metric. A lower FADE score indicates that more fog has been successfully removed and visibility has been improved in the image using the dehazing process. FADE provides a clearer evaluation of the amount of haze left in the dehazed image than other measures such as CC (Contrast Change), e (Edge Strength), r (Restoration Quality), and CNC (Comprehensive No-Reference Quality Metric). However, having a low FADE value alone does not necessarily mean that the overall dehazing effect is the best, as other factors like colour distortion and oversaturation should also be considered. It is mathematically formulated as ${\varvec{F}}{\varvec{A}}{\varvec{D}}{\varvec{E}}=\frac{1}{{\varvec{N}}}\sum_{{\varvec{i}}=1}^{{\varvec{N}}}\frac{|{{\varvec{I}}}_{{\varvec{i}}}-{{\varvec{I}}}_{{\varvec{i}},{\varvec{r}}{\varvec{e}}{\varvec{f}}}|}{\mathbf{max}\left({{\varvec{I}}}_{{\varvec{i}}}\right)}\boldsymbol{}$, where N is the total number of pixels in the image, ${{\varvec{I}}}_{{\varvec{i}}}$ is the pixel value of the image being evaluated and ${{\varvec{I}}}_{{\varvec{i}},{\varvec{r}}{\varvec{e}}{\varvec{f}}}$​ is the corresponding pixel value in the reference (original) image.

To avoid bias towards certain models, all models in the study were trained from scratch using the TensorFlow framework. This was necessary because PyTorch was the only platform that provided implementation and trained weights for most of the models. Every model was trained for 30 epochs using a portion of the RESIDE (https://sites.google.com/view/reside-dehaze-datasets/reside-standard?authuser=3D0) outdoor dataset, which included about 2000 images. It is observed that FFA Net outperforms other SOTA models. Following the comparison studies, we found that, in addition to complete reference metrics such as PSNR, no reference metric measures such as naturalness index and haze-specific metrics such as fog density are essential for a thorough examination in the dehazing field.

We first developed the hazy-KITTI dataset, whose quality we calculated using naturalness index and human opinion scores. The dataset had been a synthesised one, but the metrics indicated it would very closely replicate real world scenarios. As the dataset was generated from LiDAR sensor images, it also had some drawbacks like inconsistent haze near object edges. For KITTY dataset, special care was taken so that each model was trained until convergence, we kept the number of maximum epochs for each model at 50, and most models converged before the limit. Also, effort was taken to make the metric as close as possible to the ones the authors were able to achieve in their respective works.

After the study, we could get close performance values for most of the models, this makes our work one of the first to rigorously compare various dehazing models. After the comparative study we found that FFA net was truly a standout net even when we implemented a mini version. We also reaffirmed the fact that GMAN, GCA, and U-NET are all good variants and call be used depending on requirements and constraints. Lastly, we were able to design a net based on strong theoretical backing, which outperformed the other networks in most of the metrics. The quantitative performance of the proposed method is compared against state-of-the-art methods using several metrics. The quantitative results are tabulated in Table 5. It can be observed from this table that the proposed method achieves the best performance in terms SSIM. We have attempted to obtain the best possible results for the other methods by fine-tuning their respective parameters based on the source code released by the authors and kept the parameter consistent for all the experiments. The comparison study of dehazing models on KITTI and RESIDE datasets is presented in Tables 4 and 5 respectively.

**Table 5:** Comparison study of dehazing models on Hazy-KITTI dataset^53^.

Metrics	Hazy	LCA-Net^47^	DehazeNet^3,48^	GMAN^49^	CP^50^	U-Net^51^	FFA-Net^52^	Proposed
PSNR	15.66	18.32	20.87	24.64	22.94	22.56	27.45	31.35
SSIM	0.73	0.69	0.69	0.89	0.90	0.86	0.90	0.95
FADE	1.12	0.63	0.62	0.43	0.41	0.57	0.39	0.45
NQIE	2.75	4.36	3.92	3.09	3.21	2.73	2.54	2.79
CQIE	3.14	3.02	3.22	3.23	3.11	3.19	3.23	3.27
BLIINDS2	6.23	40.80	23.40	16.13	3.97	8.88	9.54	11.86

### Qualitative analysis

**Fig. 12:**
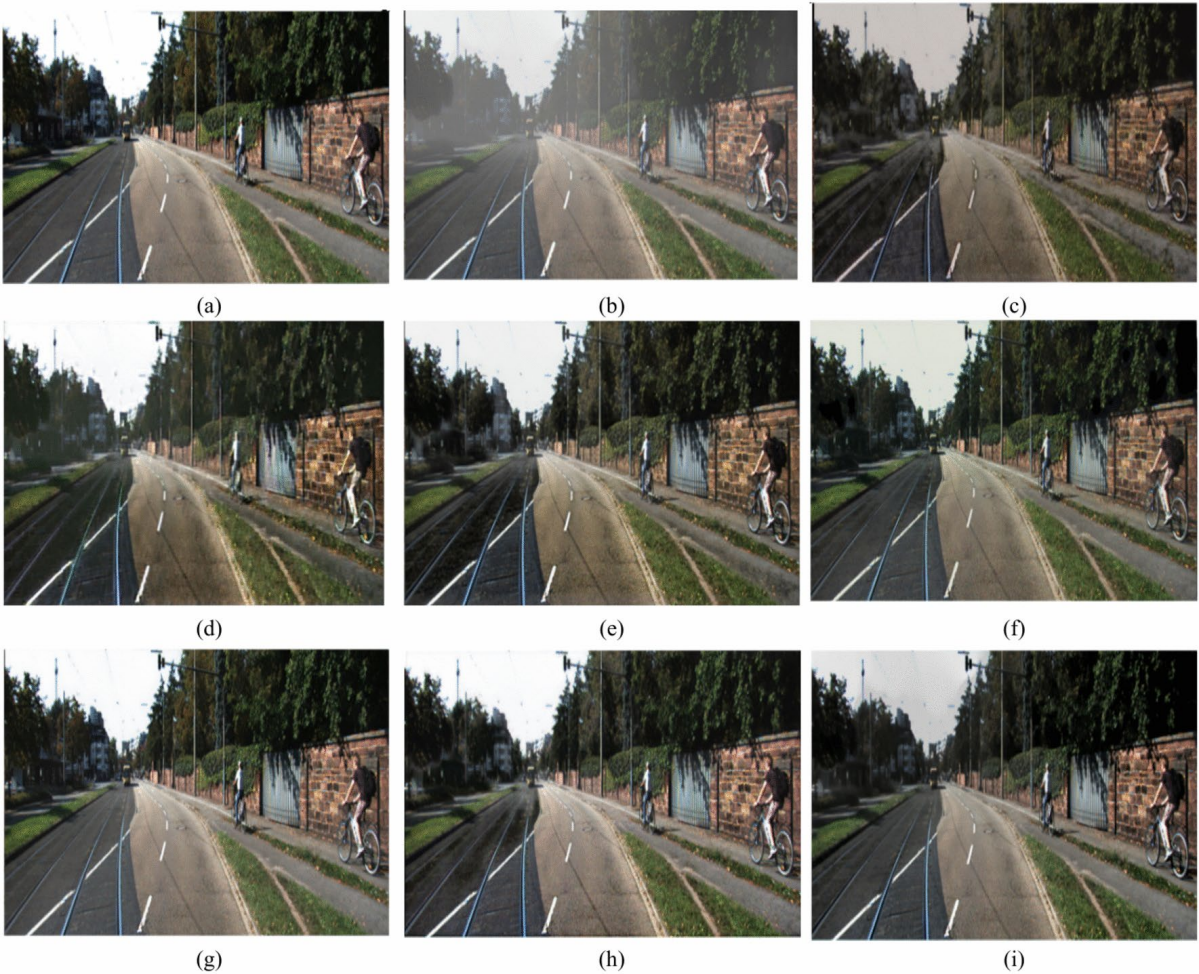
Comparison of the outputs of various models in Hazy-KITTI dataset^53^ (**a**) Ground Truth (**b**) Hazy image (**c**) LCA^47^ (**d**) Dehazenet^3,48^ (**e**) GMAN^49^ (**f**) CP^50^ (**g**) UR-Net^51^ (**h**) FFA^52^ (**i**) Proposed.

**Fig. 13:**
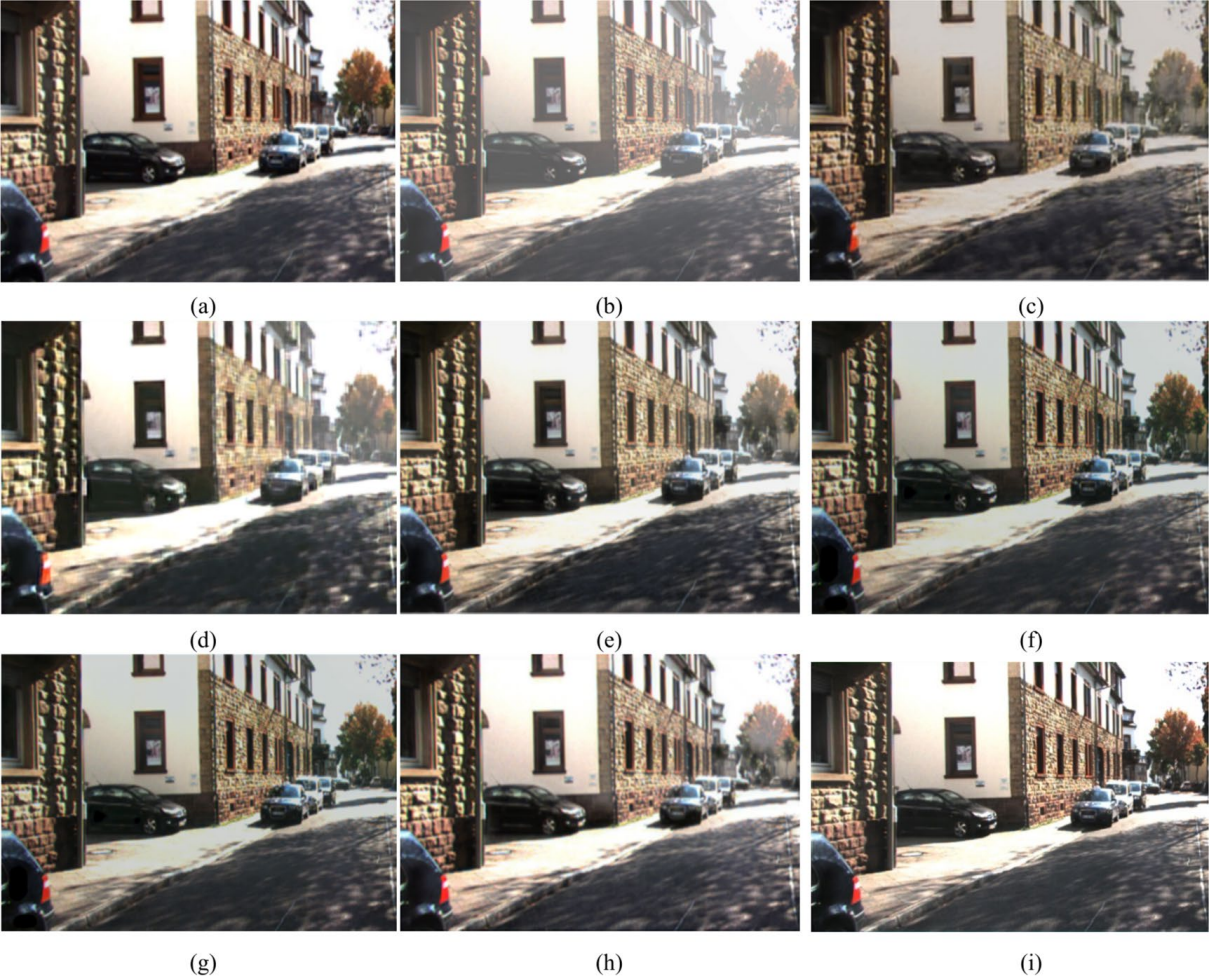
Comparison of the outputs of various models on Hazy-KITTI data set 2^53^ (**a**) Ground Truth (**b**) Hazy image (**c**) LCA^47^ (**d**) Dehazenet^3,48^ (**e**) GMAN^49^ (**f**) CP^50^ (**g**) UR-Net^51^ (**h**) FFA^52^ (**i**) Proposed.

**Fig. 14:**
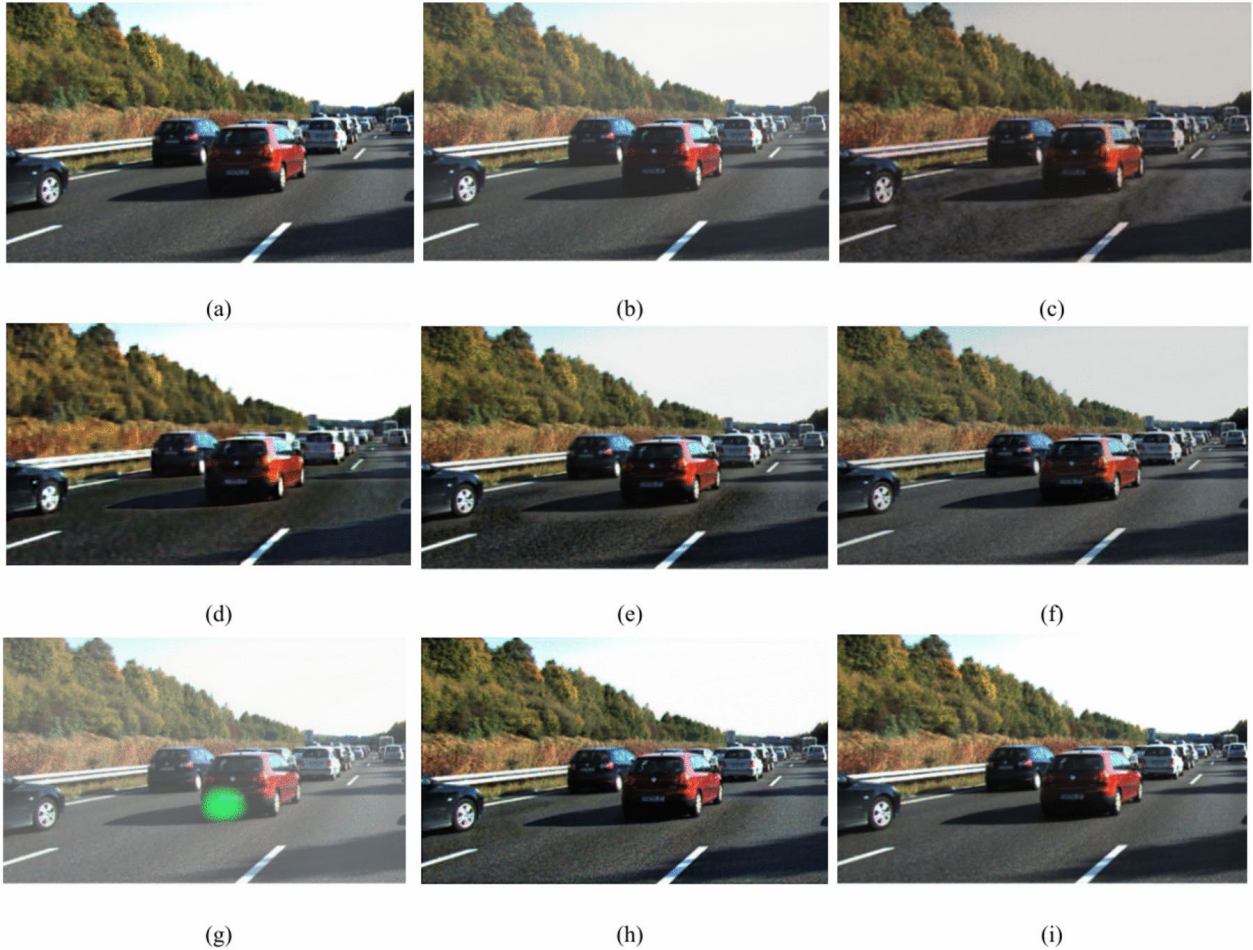
Comparison of the outputs of various models on Hazy-KITTI data set 3^53^ (**a**) Ground Truth (**b**) Hazy image (**c**) LCA^47^ (**d**) Dehazenet^3,48^ (**e**) GMAN^49^ (**f**) CP^50^ (**g**) UR-Net^51^ (**h**) FFA^52^ (**i**) Proposed.

**Fig. 15:**
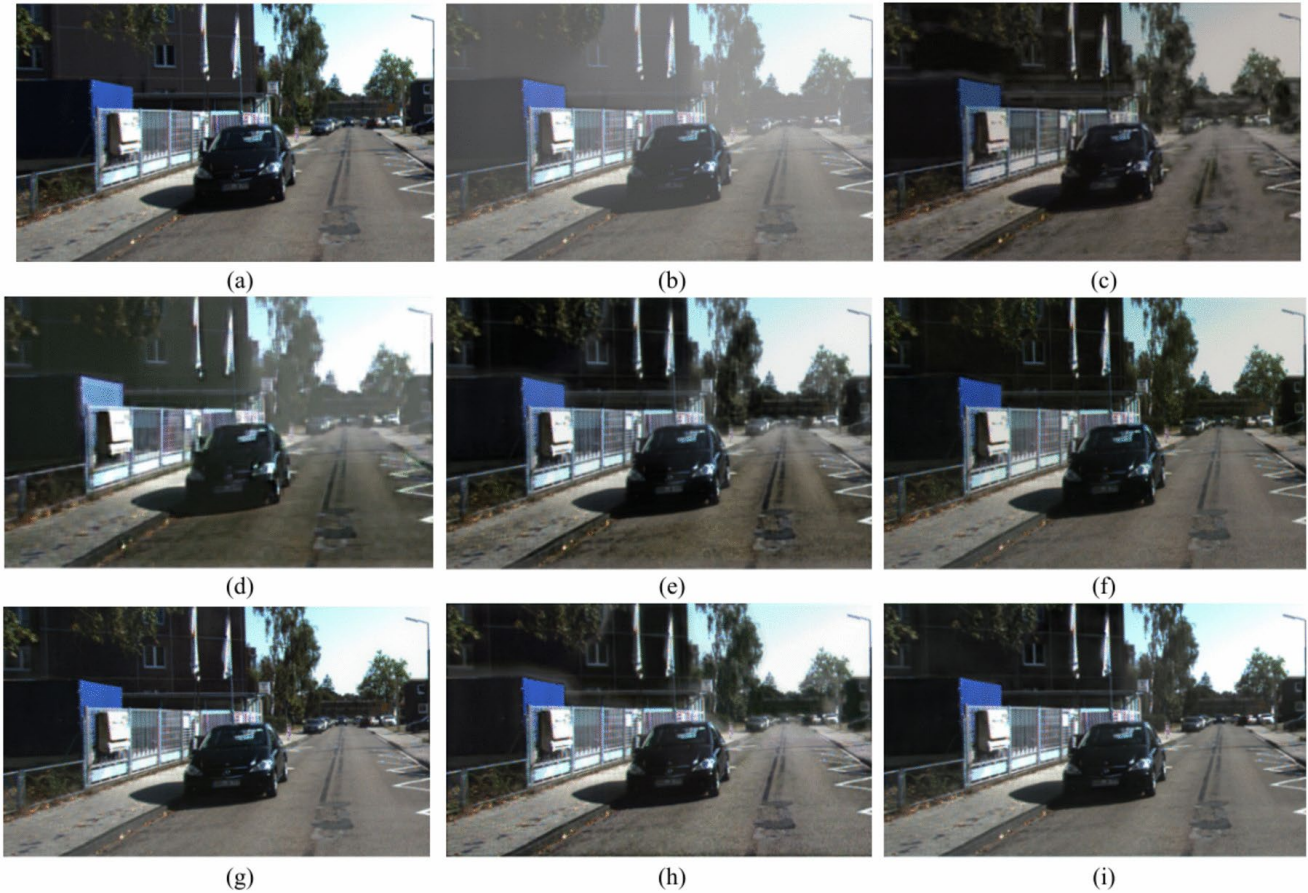
Comparison of the outputs of various models on the Hazy-RESIDE dataset 1^54^ (**a**) Ground Truth (**b**) Hazy image (**c**) LCA^47^ (**e**) Dehazenet^3,48^ ) GMAN^49^ (**g**) CP^50^ (**h**) UR-Net^51^ (**i**) FFA^52^ (**j**) Proposed.

**Fig. 16:**
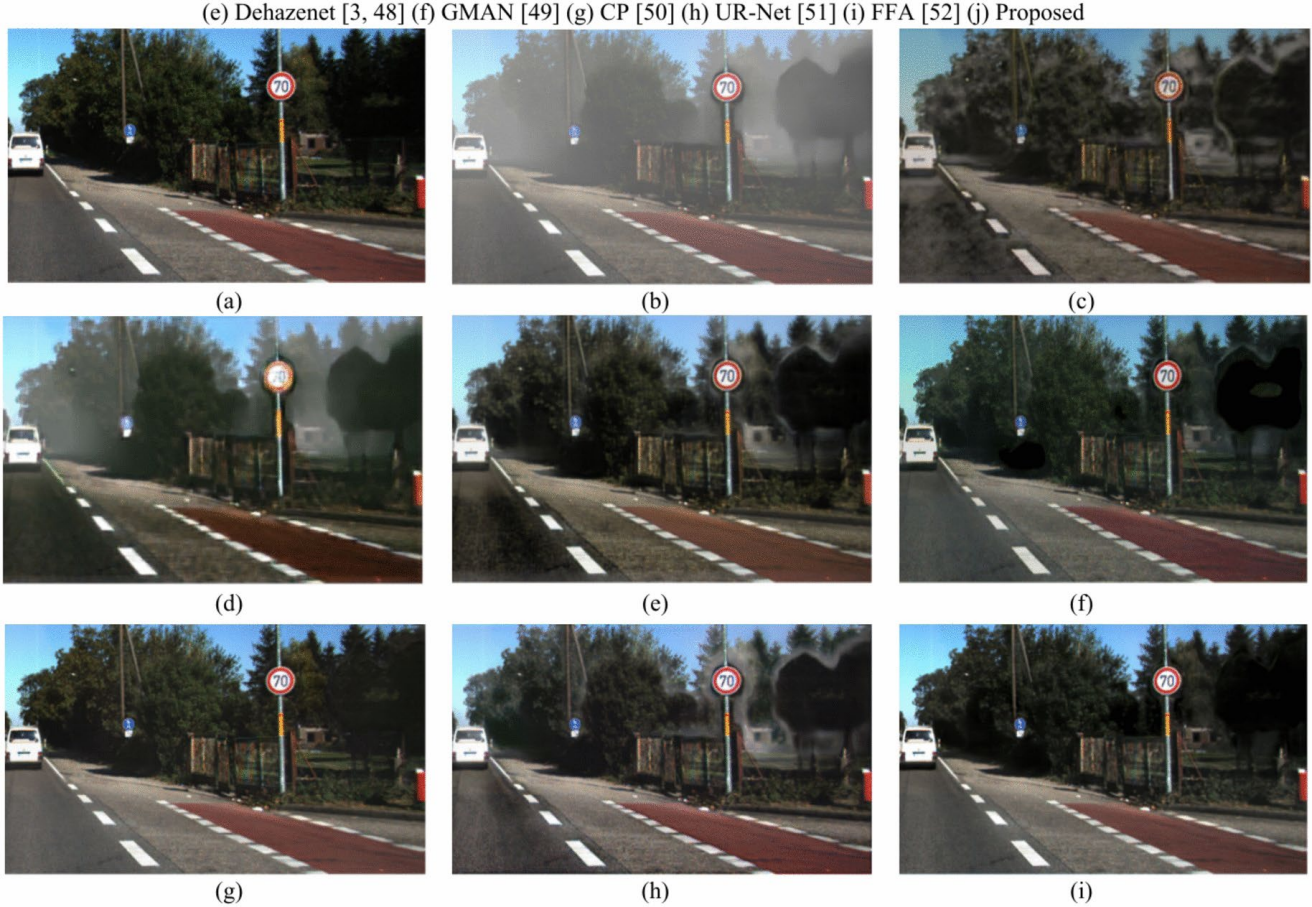
Comparison of outputs of various models on Hazy-RESIDE dataset 2^54^ (**a**) Ground Truth (**b**) Hazy image (**c**) LCA^47^ (**d**) Dehazenet^3,48^ (**e**) GMAN^49^ (**f**) CP^50^ (**g**) UR-Net^51^ (**h**) FFA^52^ (**i**) Proposed.

Our work is not limited to a dehazing net; it also provides information about atmospheric light which is directly correlated with light intensity, and it develops a transmission map in the intermediate layers, which is produced from a depth net that has been trained. As a result, it may be applied to determine the density of haze, and, in a subsequent investigation, it can be easily integrated into the study of depth correction under cloudy conditions. The comprehensive tests conducted on datasets containing difficult to interpret blurry images show that the suggested approach outperforms the state-of-the-art techniques by a considerable margin. The subjective results comparisons are presented in Figs. 12, 13, 14, 15 and 16.

The sample image of the road and the hazy image are shown in Fig. 12. Following a thorough examination of these images, we found that the most recent methods either did not manage to remove all haze or over corrected, which reduced the image’s visual appeal. While DCP eliminates haze, it additionally increases contrast in the image. However, LCA results in low quality because it retains some haze in the image. While Dehaze Net and GCA are not able to completely eliminate haze from an image, they excel LCA, GMAN, and U-Net in terms of preserving the image’s uniqueness even with reduced contrast. On the other hand, given a range of haze contents, the proposed approach and FFA can produce better dehazing results.

The example image of the road and the hazy image is shown in Fig. 13. Following an extensive review of these results, we discovered that the most current methods either failed to remove all haze or overcorrected, which lessened the image’s visual appeal. DCP removes the haze in the image but does not bring back the original colour. However, LCA produces low quality as it keeps significant portions of the image hazy. The Dehaze Net eliminates the haze, yet the distortion deteriorates the image. Although GMAN, GCA, and U-Net perform more effectively than LCA, these algorithms are unable to remove haze from images but do an improved job of maintaining the image’s uniqueness even with lowered contrast. However, better dehazing results can be achieved with the suggested approach.

Figure 14 shows the road sample image and the hazy image. After careful analysis of these results, we observed that the recent best methods resulted in either incomplete removal of haze or overcorrection which reduced the visual appeal of the image. DCP removes the haze from the image, however it fails to retain the original colour of the image. While LCA retains the haze in major portion of the image leading to poor quality. DehazeNet removes the haze but degrades the image by pixelating. GMAN, GCA cannot remove the haze from the image, however, it performs better compared to LCA. U-Net retains the haze in image and introduces the artifacts. In contrast, the proposed method and FFA can achieve better dehazing results.

On a sample of images from the KITTI dataset, Fig. 15 illustrates the results of the suggested method compared to previous state-of-the-art methods. Upon careful examination of these results, we found that the best of the the best of the most recent methods either produced inadequate inadequate haze removal or exaggerated the image, which degraded its aesthetic appeal. DCP, GMAN, DehazeNet, and LCA are unable to completely remove haze from the image. While U-Net has the ability to achieve good performance in moderate haze, colour shift frequently appears in its dehazed output. In contrast, the suggested method may achieve better dehazing for a range of haze contents. Considering the quality of the transmission maps estimated by the suggested multitask technique with those estimated by the current methods, comparable results can be observed.It is noteworthy that the quality of the dehazed images is inferior with the previous methods since they are unable to precisely measure the relative depth in each image. On the other hand, the suggested approach produces higher quality dehazing in addition to high quality transmission map estimation.

## Conclusions

In this study, we introduced ODD-Net, a hybrid deep learning architecture designed to overcome the limitations of existing image dehazing methods. By incorporating Atmospheric Light Net (A-Net) for precise atmospheric light estimation and T-Net for accurate transmission map generation, ODD-Net delivers superior dehazing performance. Our analysis of the dataset reveals that ODD-Net surpasses state-of-the-art methods such as DCP, GMAN, DehazeNet, and LCA, achieving the highest performance metrics scores and producing clearer and more accurate images. This work not only presents a comprehensive dataset but also rigorously compares various dehazing models, affirming the robustness and effectiveness of our approach. Future research could focus on developing more efficient versions of ODD-Net for real-time applications in autonomous driving and surveillance, expanding the dataset to cover diverse atmospheric conditions, and integrating additional sensor data like LiDAR or radar to enhance accuracy and reliability. These efforts will further enhance ODD-Net’s practical applicability and performance, solidifying its role as a leading solution in image dehazing.

## Data Availability

Dataset will be made publicly available after the acceptance of the paper. Code will be released via github and made available by the corresponding author on reasonable request.
